# Supplementary material for: Micronutrients absorbed via the oral mucosa reduce emotion dysregulation in 5-10-year-old children: A three-phased randomized wait-list-controlled trial
Source: PLoS One. 2024 Dec 5;19(12):e0311794. doi: 10.1371/journal.pone.0311794 (PMC11620378; doi:10.1371/journal.pone.0311794)
Supplement: S1 Table — (DOCX) [file pone.0311794.s001.docx]

**Table S1. Ingredients and Doses of the Micronutrients.**

| **Micronutrient Stick Ingredients** | **Dose per Stick** |
| --- | --- |
| Vitamin A (retinyl palmitate) | 0.03 mg |
| Vitamin C (ascorbic acid) | 10.00 mg |
| Vitamin D (cholecalciferol) | 0.60 mcg |
| Vitamin E (d-alpha tocopheryl succinate) | 4.00 mg |
| Thiamine (thiamine mononitrate) | 0.30 mg |
| Riboflavin | 0.23 mg |
| Niacin (niacinamide) | 1.50 mg |
| Vitamin B6 (pyridoxine hydrochloride) | 0.60 mg |
| Folic Acid | 0.02 mg |
| Vitamin B12 (cyanocobalamin) | 0.02 mg |
| Biotin | 0.02 mg |
| Pantothenic Acid (d-calcium pantothenate) | 0.35 mg |
| Calcium (as chelate) | 22.00 mg |
| Iron (as chelate) | 0.23 mg |
| Phosphorus (as chelate) | 14.00 mg |
| Iodine (from Atlantic kelp) | 3.40 mcg |
| Magnesium (as chelate) | 10.00 mg |
| Zinc (as chelate) | 0.80 mg |
| Selenium (as chelate) | 3.40 mcg |
| Copper (as chelate) | 0.12 mg |
| Manganese (as chelate) | 0.16 mg |
| Chromium (as chelate) | 0.01 mg |
| Molybdenum (as chelate) | 2.40 mcg |
| Potassium (as chelate) | 4.00 mg |
| Proprietary Blend: Choline Bitartrate, DL-Phenylalanine, Vanadium Chelate, Citrus Bioflavonoids, Inositol, L-Glutamine, L-Methionine, Boron Chelate, Grape Seed Extract, Ginkgo Biloba Leaf, Germanium Sesquioxide, Nickel Chelate | 27.72 mg |
| Note. Mg = milligram; mcg = microgram. |  |
