# Supplementary material for: Micronutrients absorbed via the oral mucosa reduce emotion dysregulation in 5-10-year-old children: A three-phased randomized wait-list-controlled trial
Source: PLoS One. 2024 Dec 5;19(12):e0311794. doi: 10.1371/journal.pone.0311794 (PMC11620378; doi:10.1371/journal.pone.0311794)
Supplement: S2 Table — (DOCX) [file pone.0311794.s002.docx]

**Table S3. Descriptive Information for the EMO-I for Participants at Baseline and End of Trial.**

|  | **Baseline** | | | **End of Trial** | |
| --- | --- | --- | --- | --- | --- |
|  | **ITG (*N*=24)** | | **IWLG (*N*=24)** | **ITG(*N*=22)** | **IWLG(*N*=19)** |
| EMO-I Domain | | *N* (%) | *N* (%) | *N* (%) | *N* (%) |
| Outburst Severity/Behaviors | |  |  |  |  |
| Expresses anger in an appropriate way (e.g., explains their perspective; goes to their room to cool down) | | 3 (12.5) | 3 (12.5) | 10 (45.5) | 10 (52.6) |
| Argues, whines, sulks | | 19 (79.2) | 21 (87.5) | 17 (77.3) | 16 (84.2) |
| Becomes verbally insulting, swears, shouts | | 20 (83.3) | 21 (87.5) | 12 (54.6) | 11 (57.9) |
| Threatens | | 14 (58.3) | 15 (62.5) | 5 (22.7) | 6 (31.6) |
| Slams doors, punches walls, makes a mess, destroys property | | 20 (83.3) | 19 (79.2) | 8 (36.4) | 5 (26.3) |
| Self-mutilates, bangs head, or otherwise takes it out on self | | 1 (4.2) | 3 (12.5) | 0 (0) | 0 (0) |
| Throws things | | 15 (62.5) | 19 (79.2) | 7 (31.8) | 8 (42.1) |
| Hits, kicks, bites, spits | | 13 (54.2) | 15 (62.5) | 6 (27.3) | 6 (31.6) |
| Needs physical restraint | | 5 (20.8) | 4 (16.7) | 3 (13.6) | 1 (5.3) |
| Frequency | |  |  |  |  |
| Never | | 0 (0) | 0 (0) | 0 (0) | 3 (15.79) |
| Rarely | | 2 (8.3) | 0 (0) | 14 (63.6) | 5 (26.3) |
| Several times a month | | 5 (20.8) | 3 (12.5) | 1 (5.6) | 4 (21.1) |
| Weekly | | 8 (33.3) | 5 (20.8) | 3 (13.6) | 3 (15.8) |
| At least 3 times per week | | 5 (10.8) | 9 (37.5) | 3 (13.6) | 3 (15.8) |
| Daily | | 4 (16.7) | 7 (29.2) | 1 (4.6) | 1 (5.3) |
| Duration | |  |  |  |  |
| A few minutes | | 3 (12.5) | 2 (8.3) | 13 (59.1) | 11 (57.9) |
| Up to 15 minutes | | 6 (25.0) | 9 (37.5) | 8 (35.4) | 5 (26.3) |
| Up to half an hour | | 7 (29.2) | 8 (33.3) | 0 (0) | 2 (10.5) |
| Up to an hour | | 7 (29.2) | 4 (16.7) | 1 (4.6) | 1 (5.3) |
| Up to half a day | | 1 (4.2) | 1 (4.2) | 0 (0) | 0 (0) |
| Location | |  |  |  |  |
| At home/with parents | | 18 (75.0) | 18 (75.0) | 20 (90.1) | 14 (73.7) |
| At school | | 0 (0) | 0 (0) | 0 (0) | 0 (0) |
| Both home and school | | 3 (12.5) | 1 (4.2) | 2 (9.1) | 3 (15.8) |
| Home, school, public | | 3 (12.5) | 4 (16.7) | 0 (0) | 2 (10.5) |
|  | | | | | |
