# Supplementary material for: Micronutrients absorbed via the oral mucosa reduce emotion dysregulation in 5-10-year-old children: A three-phased randomized wait-list-controlled trial
Source: PLoS One. 2024 Dec 5;19(12):e0311794. doi: 10.1371/journal.pone.0311794 (PMC11620378; doi:10.1371/journal.pone.0311794)
Supplement: S3 Table — (DOCX) [file pone.0311794.s003.docx]

**Table S4. Recorded Adverse Events during the RCT Phase.**

| **Adverse events** | **ITG (*N*=24) (%)** | **IWLG (N=24) (%)** | ***p*-value** |
| --- | --- | --- | --- |
| Dry mouth | 3 (12.5) | 0 (0) | .202 |
| Drowsiness | 2 (8.3) | 5 (20.8) | .528 |
| Insomnia | 8 (33.3) | 12 (50) | .592 |
| Blurred vision | 1 (4.1) | 0 (0) | .312 |
| Headache | 6 (25) | 12 (50) | .040* |
| Constipation | 3 (12.4) | 6 (25) | .468 |
| Diarrhea | 3 (12.5) | 2 (8.3) | .600 |
| Increased appetite | 4 (16.7) | 3 (12.5) | .795 |
| Decreased appetite | 2 (8.3) | 4 (16.7) | .553 |
| Nausea and vomiting | 4 (16.7) | 5 (29.8) | .669 |
| Problems with urination | 0 (0) | 3 (12.5) | .202 |
| Palpitations | 2 (8.3) | 0 (0) | .352 |
| Feeling light-headed on standing | 0 (0) | 3 (12.5) | .074 |
| Feeling like the room is spinning | 0 (0) | 2 (8.3) | 0.149 |
| Sweating | 0 (0) | 4 (16.7) | .037* |
| Increased body temperature | 2 (8.3) | 7 (29.2) | .014 |
| Tremor | 0 (0) | 0 (0) | 1 |
| Disorientation | 0 (0) | 0 (0) | 1 |
| Yawning | 5 (20.8) | 7 (29.2) | .548 |
| Weight gain | 3 (12.5) | 0 (0) | 0.202 |
| Rash | 1 (4.1) | 0 (0) | .312 |

Note. * = *p* < .05
