# Supplementary figures and images for: Micronutrients absorbed via the oral mucosa reduce emotion dysregulation in 5-10-year-old children: A three-phased randomized wait-list-controlled trial
Source: PLoS One. 2024 Dec 5;19(12):e0311794. doi: 10.1371/journal.pone.0311794 (PMC11620378; doi:10.1371/journal.pone.0311794)

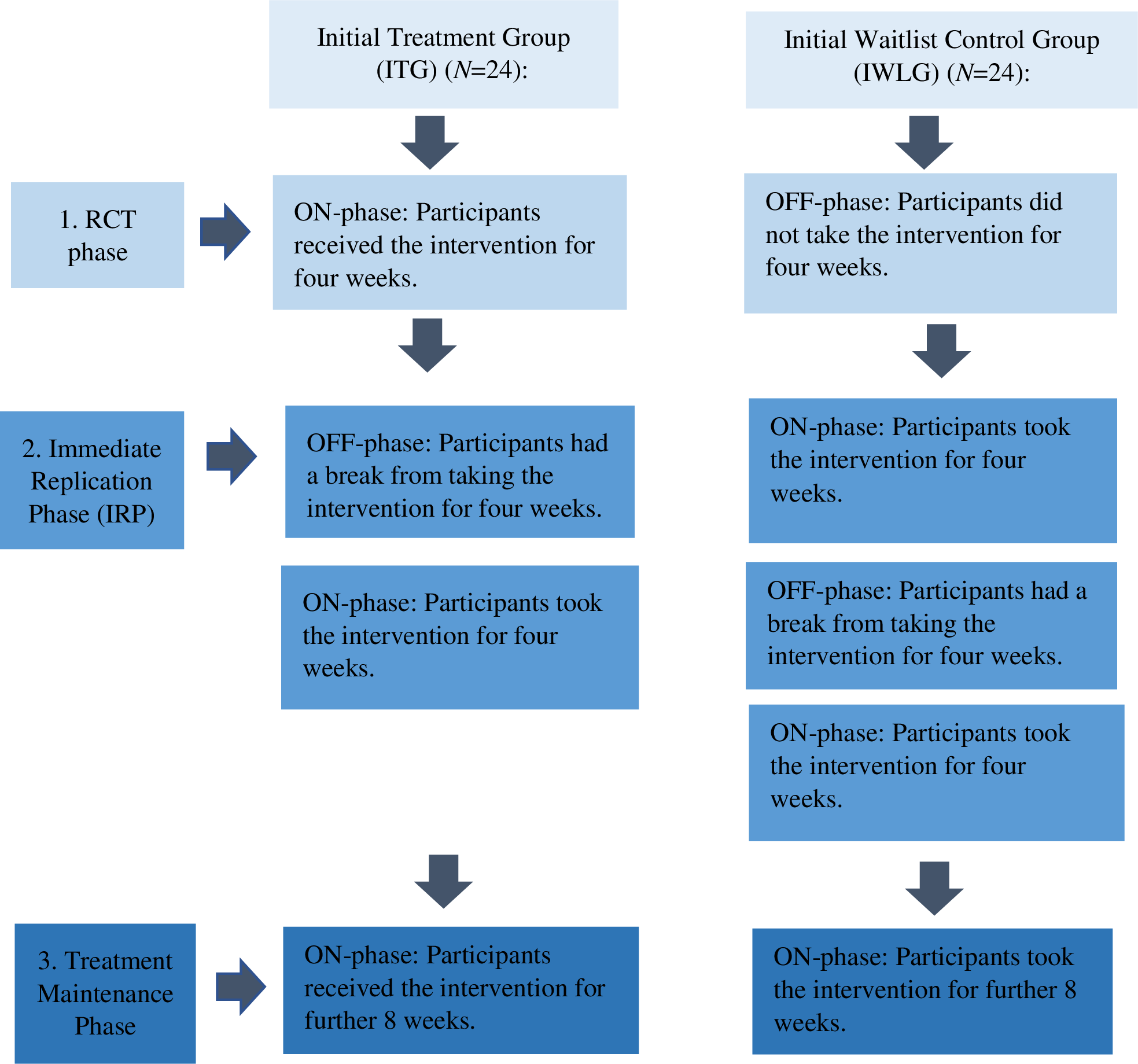

Supplement: S1 Fig — (TIF) [file pone.0311794.s004.tif]
