# Supplementary material for: Micronutrients absorbed via the oral mucosa reduce emotion dysregulation in 5-10-year-old children: A three-phased randomized wait-list-controlled trial
Source: PLoS One. 2024 Dec 5;19(12):e0311794. doi: 10.1371/journal.pone.0311794 (PMC11620378; doi:10.1371/journal.pone.0311794)
Supplement: S2 Appendix — (DOCX) [file pone.0311794.s006.docx]

**Protocol for Micronutrients and Emotion Dysregulation in Children: A Two-arm Withdrawal Single-Case Design**

**Principal Investigators: Nurina Maria Katta/Prof. Julia Rucklidge**

Te Puna Toiora: Mental Health and Nutrition Research Lab

University of Canterbury

Private Bag 4800,

Christchurch 8140, NZ.

Email: [nurina.katta@pg.canterbury.ac.nz](mailto:nurina.katta@pg.canterbury.ac.nz)

Phone: 022 010 5522

Contents

[Background](#_Toc17724173) 3

[Aims and Objectives](#_Toc17724173) 9

[**Method**](#_Toc17724174) 10

[Study design](#_Toc17724175) 10

[Assessment Tools](#_Toc17724176) 13

[Statistical Analysis](#_Toc17724177) 16

[Anticipated costs](#_Toc17724178) 18

[Resources](#_Toc17724179) 18

[Timetable](#_Toc17724180) 18

[Ethics](#_Toc17724181) 18

[Safety protocol related to visits at the University Lab](#_Toc17724182) 18

[Cultural sensitivity](#_Toc17724183) 20

[Research site](#_Toc17724183) 21

[Investigators](#_Toc17724184) 21

[Risk Management](#_Toc17724185) 21

[Data ownership](#_Toc17724186) 21

[Conflicts of interest](#_Toc17724187) 22

[**References**](#_Toc17724188) 23

[**Appendices**](#_Toc17724189) 30

[Appendix A: Lightning Stiks active intervention ingredient list](#_Toc17724190) 30

[Appendix B: Schedule of Events](#_Toc17724191) 31

# Background

Emotion dysregulation among children can frequently be observed in different contexts, interfering with various important aspects of life, including general development, social skills, and family dynamic in the home. The following paragraphs introduce the concept of emotion regulation, and explore the link of emotion dysregulation to other issues such as irritability, stress, inattention, and hyperactivity/impulsivity. Further, the increasing role of vitamins and minerals as an intervention for emotion dysregulation and irritability is discussed by means of research from the past decade, illustrating the relevance of nutritional research as a treatment option. Lastly, the aims and objectives of the proposed study are presented.

Competent emotion regulation is defined as the “extrinsic and intrinsic processes that serve to monitor, evaluate, and modify emotional reactions” (Thompson, 1994, p. 27-28), referring to the effective management of behaviours following experiences in the environment that trigger emotions. Competent emotion regulation does not only foster empathy but also enables one to know the difference between internal emotional experiences and the external expression of emotions, behave socially appropriate (Thompson, 2019), and adapt to external stressors by identifying, understanding, and integrating emotional information (Garnefski et al., 2007; Saarni, 1984; Terwogt & Stegge, 1995; Thompson, 1994). It is a complex concept that depends on multiple aspects, including the person’s developmental status, subjective perceptions, cultural norms and values, and past experiences (Kopp, 1982; Saarni, Campos, Camras, & Witherington, 2007). When children are five, they are usually competent to appropriately regulate their emotions and behaviour (Carlson & Wang, 2007), and this has a positive impact on various developmental factors such as social skills, language, and cognitive abilities (Kamphaus, 1987). For example, in preschool children, competent emotion regulation has been associated with superior social behaviour, social competence, and abilities to adjust to the environment, as well as being liked by peers and teachers (Denham et al., 2003).

Because competent emotion regulation helps us to effectively face challenges, adequate emotion regulation skills are required for successful coping with stressful situations or life events (Saarni et al., 2007; Wong et al., 2006), which might be a reason why emotion regulation is frequently associated with stress. The overlap between emotion regulation and stress might also be explained by common physiological mechanisms, as they both involve activation of the hypothalamic–pituitary–adrenal (HPA) axis (Wang & Saudino, 2011). Specifically, Zimmermann and Stansbury (2004) showed that competent emotion regulation acts as a predictor for lower levels of cortisol, a stress hormone, which is initiated by triggering the HPA axis. In addition, evidence suggests that the prefrontal cortex is activated both during stress and emotion regulation processing (Beauregard, Lévesque, & Bourgouin, 2001; Ochsner, Bunge, Gross, & Gabrieli, 2002), indicating that there is a neuroanatomical overlap in the brain areas linked to both states.

Whereas competent emotion regulation has many beneficial outcomes, emotion dysregulation is associated with a series of adverse effects, including psychosocial issues, anxiety, poor academic achievement, and behavioural escalations in the home (Zeman, Cassano, Perry-Parrish, & Stegall, 2006). Emotion dysregulation is operationally defined as “a pattern of emotional experience and/or expression that interferes with appropriate goal-directed behaviour” (Beauchaine, 2015, p. 876), with goal directed behaviour referring to competent emotion regulation and its associated functions as described above (Thompson, 2019). In contrast to competent emotion regulation, it is the maladaptive management of emotional responses, and has been shown to damage social relationships and can possibly lead to isolation and psychosocial stress (Eisenberg et al., 1993; Stansbury & Gunnar, 1994). These negative outcomes are highlighted by the longitudinal Dunedin cohort study, which revealed that children with uncontrolled temperament at age 3 were twice as likely to develop a gambling disorder at age 21 and 32 relative to children classified as having a well-adjusted temperament (Slutske et al., 2021). The same study showed that better self-control skills in children predicted improved physical health, reduced substance use disorders, and less criminal offending in later life (Moffitt et al., 2014).

Emotion dysregulation is a highly prevalent phenomenon, particularly in children and young people, and frequently linked to psychiatric disorders (Pylypow et al., 2020). In healthy youth, prevalence rates range from 3% to 20% (Brotman et al., 2006), which increase up to 33% in individuals with psychiatric conditions (Stringaris, 2011). Further, it is increasingly acknowledged as a prominent feature of Attention-Deficit/Hyperactivity Disorder (ADHD) (Retz, Stieglitz, Corbisiero, Retz-Junginger, & Rösler, 2012), a common psychiatric disorder characterized by problems with hyperactivity/impulsivity and inattention (American Psychiatric Association, 2013). Depending on comorbid diagnoses, up to 90% of children with ADHD also struggle with emotion dysregulation (Mick, 2005). Similar to emotion dysregulation, ADHD is linked to adverse long-term consequences, such as poor academic performance, impaired social relationships, and increased substance abuse (Barkley, 2006).

Although emotion dysregulation is common, it is frequently examined within various psychiatric disorders rather than as an independent construct, possibly impacting the establishment of treatment strategies (Brotman et al., 2017). So far, treatment for emotion regulation has included psychopharmacologic medication; particularly, in one placebo-controlled RCT, the selective serotonin reuptake inhibitor (SSRI) fluoxetine improved aggression and irritability in adults relative to a placebo (Coccaro et al., 2009). In another placebo-controlled RCT with youth with severe and chronic irritability, a higher response-to-treatment-rate was seen in the participants who received an SSRI in combination with a stimulant (citalopram and methylphenidate) compared to a placebo combined with a stimulant only (methylphenidate) (Towbin et al., 2020); however, the overall response rates were modest (35% and 6% respectively).

Psychiatric medication in the form of stimulants are often prescribed as the main form of treatment for individuals with ADHD, who frequently have problems with emotion dysregulation (Barbaresi et al., 2014). However, although research has shown that stimulant medications are an effective intervention in the short term (Biederman et al., 2004), issues have been raised regarding their long-term benefits (Advokat, 2010, Currie et al., 2014; Molina et al., 2009). Additionally, the literature shows that while stimulant medication may benefit some ADHD-related symptoms, it frequently fails to improve emotion dysregulation (Shaw et al., 2014; Posner et al., 2014), particularly in younger people (Posner et al., 2014), indicating that emotion dysregulation might be a separate construct to ADHD. In addition, several concerning side effects have been observed with stimulant medication including weight reduction and suppression of growth (Thomas et al., 2013), effects on brain development depending on the age when the stimulants are used (Andersen, 2005), nausea, cardiovascular issues, insomnia, and agitated behaviours (Baumgaertel, 1999). Overall, the current evidence on psychiatric medication as a treatment for emotion regulation in youth is sparse and suggests limited benefits, posing a need to future research in this area (Towbin et al., 2020; Brotman et al., 2017).

Alternatively, behavioural interventions, particularly Cognitive Behavioural Therapy (CBT), have shown to be an effective intervention for young individuals who have issues with anger and aggression (Brotman et al., 2017). CBT helps such children learn how to minimize maladaptive behaviour by considering alternative and adaptive ways to think about and react to triggering situations (Kazdin, 2010). However, although behavioural therapies for young people are frequently suggested, there is a lack of guidelines on how to apply evidence-based treatment strategies (Daley et al., 2018) and a shortage of trained clinicians and specialist services (Miller et al., 2014), which can interfere with effective treatment delivery.

Due to some of the downsides of the current treatment options as well as the fact that dysregulation of emotions is difficult to treat and does not appear to respond as well to some of the current treatment options, other treatments have been investigated. In particular, there has been an increase in investigations of the role of nutrition in mental health, and evidence has established that the intake of healthy food, such as fruit, vegetables, legumes, and healthy fats, is linked to improved mental wellbeing (Jacka et al., 2017; Parletta et al., 2018). However, given the research that has uncovered that the nutrient density of our foods and our soils has been progressively decreasing (Rucklidge & Kaplan, 2021), and may become more so, as a consequence of climate change (FAO, 2017), eating foods that are considered nutritionally dense might no longer have the desired beneficial effects on our mental health, making it important to seek alternative ways of increasing nutrient availability in our brain. Supplementing food consumption with nutrients might be one way to do so, and over the past decades, there has been an increasing number of studies investigating the role of consuming additional nutrients in the form of nutritional supplements on mental wellbeing.

Initially, researchers investigated single rather than multinutrient interventions in the context of mental health issues; however, due to overall disappointing results, the focus is increasingly on combined nutrient treatments, including broad-spectrum multinutrient supplements that contain the full array of essential vitamins, minerals, and amino acids. Reasons for this shift include the fact that people tend to have multiple nutrient deficiencies rather than just one, and the evidence shows that multiple nutrient deficiencies are highly prevalent across the globe, with approximately two billion people experiencing some form of deficiency (Bailey et al., 2015). In fact, treating deficiencies with a single supplement can have adverse effects, as it can lead to imbalances in other nutrients (Mertz, 1994). Further, vitamins and minerals do not exert their effects on the brain separately but work together with other vitamins, minerals, and amino acids, challenging the initial ‘one disease-one nutrient’ approach (Mertz, 1994).

To date, various theories have been developed that aim to explain how micronutrients exert their positive effects on psychiatric symptoms. Firstly, micronutrients are an essential cornerstone of the synthesis of some of the neurotransmitters that are involved in mood, such as dopamine and serotonin, particularly, vitamin B9 (folate), B12, and B6, as well as the minerals iron, copper, and molybdenum (Stough et al., 2011). Specifically, creation of dopamine and serotonin involves multiple metabolic processes that utilize enzymes and co-enzymes that require various vitamins and minerals as cofactors. If vitamins and minerals are not available, enzymes and co-enzymes cannot function properly, impairing dopamine and serotonin production and potentially interfering with mental wellbeing (Rucklidge & Kaplan, 2013).

Further, micronutrients are essential for the functioning of the mitochondria, the power plants for neurons and glia, that are crucial for various physiological processes for brain functioning, including energy production, metabolic processes, and neuronal communication (Stough et al., 2011). Evidence suggests that impaired mitochondrial functioning might be involved in bipolar disorder, ADHD, and Autism Spectrum Disorder (Rossignol & Frye, 2012; Russel et al., 2006; Young, 2007), and although it is not yet well established, vitamin A, C, and K might support mitochondrial functioning, supporting optimal brain functioning (Rucklidge & Kaplan, 2013).

These beneficial effects are underlined by recent evidence, illustrating the positive effect of broad-spectrum micronutrients on a variety of mental conditions and psychological symptoms, including emotion dysregulation and irritability (Rucklidge et al., 2019; Rucklidge et al., 2014; Julia & Kaplan, 2013). For instance, Rucklidge et al. (2018) conducted a double-blinded RCT with 93 7-to-12-year-old children with ADHD, with some children having severe problems with emotional dysregulation and aggression. A clinician rating of global functioning (based on consideration of parent, teacher and child reports) showed that nearly double the number of children in the micronutrient group were identified to be ‘much’ to ‘very much’ improved relative to the placebo group (47% versus 28%). In comparison with the placebo group, children who took the micronutrients showed a greater improvement in aggression (*d* = 0.52), emotionally dysregulated behaviour (*d* = 0.66), dysregulated mood (*d* = 0.35), conduct problems (*d* = 0.48), and problem behaviour based on the parent- (*d* = 0.41) and teacher-rated (*d* = 0.45) questionnaires, although it is important to note that not all the group differences were statistically significant.

Further, in an open label, on-off-on-off (reversal design) study, 14 children with a diagnosis of ADHD between 8 and 12 years old were assessed over a period of 6 months. In particular, children were given micronutrients for 8 weeks, then the treatment was withdrawn for 4 weeks, then reintroduced for 8 weeks, and withdrawn again for 4 weeks (Gordon et al., 2015). In the treatment phases, participants showed a large and statistically significant improvement across a variety of symptoms, including issues with social behaviour and conduct problems, which are symptoms frequently related to emotion dysregulation. In addition, over two-thirds of the participants were identified as ‘much’ or ‘very much’ improved after the second treatment phase. This beneficial effect was reversed when the treatment was withdrawn, which illustrates the potential benefits of micronutrients on behavioural regulation.

To illustrate the importance of nutrition in psychiatric symptoms not only in children but also adults, in an open-label pilot study, 14 adults between the age of 28 and 55 with diagnoses of both severe mood dysregulation and ADHD were given micronutrients for 8 weeks (J. Rucklidge, Taylor, & Whitehead, 2011). Data revealed that the participants experienced moderate to large statistically and clinically significant benefits across emotional symptoms, such as mood, mania, emotional lability, as well as hyperactivity/impulsivity and inattention (*d* = 0.66-1.96). These findings illustrate that micronutrients may represent an alternative treatment path for those who struggle with emotion dysregulation and irritability that needs to further be explored.

As mentioned above, emotion regulation/emotion dysregulation is frequently associated with stress, and stress is linked to various adverse effects, including the development of cardiovascular disease, atherosclerosis, oxidative stress, and inflammatory reactivity (Huang, Webb, Zourdos, & Acevedo, 2013), as well as poorer mental wellbeing, and academic underachievement (Myers et al., 2012). As an attempt to reduce stress as well as trial a new delivery modality for the micronutrients, an unpublished randomized, double-blinded trial conducted in Te Puna Toiora investigated the effect of this delivery modality on stress and mental wellbeing in UC students. The product tested was powdered multinutrient straws called EMPowerplus Lightning Stiks^TM^, which use Direct-to-Mouth technology in which the multinutrients are absorbed through the tissues of the mouth. This different modality means that the dose of the micronutirents is much lower than has been previously used in other research on broad spectrum micronutrients. Indeed, all doses are well below the recommended Dietary Allowance (RDA) and none are given in a dose above the Upper Limit (UL). None of the doses are given at a medicinal level. The Lightning Stiks^TM^ consist of a blend of 36 ingredients, including vitamins, minerals and amino acids, called EMPowerplus, and previous research has shown that this formula (taken in capsules) can improve stress, anxiety, and mood in adults as well as in children (J. J. Rucklidge et al., 2012; J. J. Rucklidge & Blampied, 2011; Sole, Rucklidge, & Blampied, 2017; Stough et al., 2011). Preliminary data analysis of this trial (STAR trial - not yet published) revealed that the Lightning Stiks^TM^ reduced stress in students more rapidly relative to the placebo group, although the group difference was not statistically significant (*d* = 0.39, *p* = .108). Additionally, the multinutrient group displayed a greater beneficial impact on emotion regulation and irritability compared to the placebo group (*d* = 0.54, *p* = .024), as well as reduced symptoms of anger (*d* = 0.62, *d* = .011).

Irritable children who struggle with emotion dysregulation, anger management, and stress coping can be found across all ages and display issues well-known to mental health practitioners and psychologists; as such, addressing the problems displayed by these children is important in future research. Anecdotal reports suggest that the effects of the Lightning Stiks^TM^ are similar to the capsule version, with many users reporting feeling calmer, happier, and more focused. Additionally, and aligning with the findings of the STAR trial, anecdotal reports of children in the community who have taken them suggested that the nutrients were well tolerated with reports of improvement in emotion dysregulation in some of the children, showing that the Lightning Stiks might be a suitable treatment for emotion dysregulation in children.

One recent study study within Te Puna Toiora researched the impact of micronutrients on children with traumatic brain injury, and for these children, taking the micronutrients (as capsules or powder) was a major struggle due to the unpleasant taste and smell. In addition, children frequently struggle with swallowing numerous capsules; a study in 2008 found that 30%-40% of parents had children (aged 0-26 years) who had difficulties with swallowing a standard sized pill, and more than 50% were unable to swallow it at all (Polaha et al., 2008). These findings underline that the Lightning Stiks^TM^ might be a new, more convenient way for children to take micronutrients.

Further, as mentioned, safety concerns associated with the doses of the micronutrients present in the Lightning Stiks^TM^ are unlikely because the nutrient doses of each ingredient are below the RDA. This low risk is supported by the findings of the STAR trial (not yet published), showing that only a small number of side effects were present in the study (drowsiness and reduced appetite), and these were temporary and disappeared during the duration of the trial. Finally, by improving emotion regulation skills, stress coping mechanisms might be improved as well, minimising the negative impact of stress on the individual.

Overall, evidence suggests that micronutrients may have a beneficial effect on emotion dysregulation, irritability, and stress. Given that emotion dysregulation in children is a common phenomenon which does not only affect the child but also the family, teachers, and social relationships, it is an issue that needs to be addressed in future research. Current treatments for emotion dysregulation show limitations in efficacy and implementation, and as such, future research needs to establish feasible and easy-to-implement interventions that can be put in place. Nutritional interventions present as non-invasive and have a low incidence of side effects that are usually mild and temporary. Further, because the Lightning Stiks^TM^ are available in multiple flavours and do not have the usual unpleasant taste and smell that multinutrient pills have, they might be a convenient alternative treatment for children, representing the foundation for the proposed study.

## Aims and Objectives

The proposed study aims to investigate whether the Lightning Stiks^TM^ are an effective short- and long-term treatment for children between 5 and 10 years of age who struggle with emotion dysregulation and irritability. It will be the first independent explorative study to investigate the feasibility and effectiveness of the Lightning Stiks^TM^ as an intervention for children with emotion dysregulation.

**Hypotheses:**

1. The Lighting Stiks^TM^ will be found to be an acceptable and effective intervention to reduce irritability, behavioural- and emotional dysregulation in 5-10 year-old children.

2. The beneficial effect of the micronutrients will be present during the phases where participants take the micronutrients, and diminish during phases where the treatment is withdrawn.

3. There will be differences in the first month between the two groups in emotion dysregulation when group 1 will be taking the nutrients and group 2 will not (see design), with group 1 showing better emotion regulation than group 2.

4. There will be differences in the second month between the two groups in emotion dysregulation when group 2 will be taking the micronutrients and group 1 will not, with group 2 showing better emotion regulation than group 1.

5. There will be differences in the third month between the groups in emotion dysregulation when group 1 will be taking the micronutrients and group 2 will not, with group 1 representing better emotion regulation than group 2.

6. During the final eight weeks of the trial, when both groups will take the nutrients simultaneously, both groups will display similar competencies in emotion regulation.

7. It is expected that the improved behaviour in children will reduce the parents’ symptoms of stress, potentially having an additional beneficial effect on the children’s behaviour.

8. The Lightning Stiks^TM^ will be found to be a safe intervention that has a low incidence of side effects which will be mild in severity and only last temporarily, and yields high consumer satisfaction in the participants.

9. The longer the participants take the Lightning Stiks^TM^ continuously at a time, the greater the beneficial effect on emotion dysregulation.

# Method

## Study design

The proposed study will use a two-arm withdrawal open-label design, following the advice of Hersen and Barlow (1976) on how to evaluate the effect of drug interventions on behaviour using a single-case approach. In particular, the study will have two groups, and participants will be randomized in equal numbers to either group 1 or group 2 by means of a randomized block procedure.

There will be five study phases:

1. Study period 1: Online screening and baseline assessment: Participant children will be screened for eligibility (see below) by providing information regarding their demographics as well as completing measures assessing emotion dysregulation and irritability. If they are eligible, they and their parent(s) will meet with the study coordinator Nurina Katta at Te Puna Toiora during the week before the intervention period starts. During that meeting, eligibility will be checked, questions about the research answered, consent obtained, and the participant will complete a baseline assessment. In case the participants cannot be met in person (e.g., due to COVID-19 or because they live outside of Christchurch), meetings will be held and consent conducted over the phone or online (via Skype/Facetime/Whatsapp/Zoom). Consent forms can be uploaded and emailed/mailed back to Te Puna Toiora. Once consent is given, baseline data will be collected by means of an online survey (Qualtrics) including various psychometric measures (see below). Participants in group 1 will be given one month’s supply of Lightning Stiks^TM^ immediately after the baseline assessment. If participants cannot be seen face-to-face, the Lightning Stiks^TM^ will be delivered by courier. Those randomised to group 2 will have a delayed 4 week start (see Figure 1)
2. Study period 2: Group 1 will start taking the intervention for 4 weeks (ON-phase). Every two weeks, the parents will fill out various psychometric questionnaires assessing their children’s emotion dysregulation and irritability (see measures below), other symptoms linked to ADHD, sleep, diet, mood, anxiety and stress. Group 2 will be in a delayed baseline phase during study period 1, but will be monitored by answering the same psychometric questionnaires as group 1 every two weeks.
3. Study period 3: The intervention in group 1 will be withdrawn for four weeks (OFF-phase), whereas group 2 starts taking the Lightning Stiks^TM^ (ON-phase). Both groups answer the same questionnaires as before every two weeks.
4. Study period 4: Group 2 stops taking the interventions (OFF-phase) whereas group 1 starts taking them for another 12 weeks (ON-phase). Both groups answer the same questionnaires as before every two weeks.
5. Study period 5: When Group 1 has been taking the Lightning Stiks^TM^ for four weeks, Group 2 starts taking the multinutrient intervention for another 12 weeks and will answer questionnaires every two weeks.

Every four weeks, the children’s parents and children will meet with the study coordinator at *Te Puna Toiora* to complete various psychometric questionnaires assessing their children’s behaviour, including hyperactivity/impulsivity, inattention, emotion dysregulation, irritability, sleep, social relationships, diet, and side effects. If participants cannot be met in person, the meeting will be held via Zoom. Both the child and the parent will answer the questions of the Measure it Yourself Medical Outcome Profile (MYMOP) (see below) jointly; otherwise, they will answer the questionnaires on their children’s or their own behalf. In addition, questionnaires assessing emotion dysregulation and irritability will also be emailed to another adult who is close to the child, such as a grandparent, aunts/uncles, or teachers, twice over the period whole study period. Please find a diagram with all study phases below.

#### **Participant recruitment**

Participants will be recruited nationally across New Zealand, mainly by self-referrals following online advertisement on social media. Specifically, recruitment will take place through advertisement on Facebook (via the Mental Health and Nutrition Research Lab Te Puna Toiora account, the University of Canterbury Student Association (USCA) noticeboard), and Instagram (via the Te Puna Toiora account). Further, advertisement posters and brochures will be distributed around the UC campus, with local General Practitioners and mental health service providers. To increase Māori participation in the study, efforts will be made to engage with Māori service providers.

*Sample size*

Forty participants will be sought for the study, split 20:20 between group 1 and 2 . This number of participants is considered a sufficient sample size to determine whether the Lightning Stiks^TM^ are successful in reducing emotion dysregulation with sufficient power. This is a reasonable expectation as it allows for an adequate number of successful replications between participants, following the What Works Clearinghouse Standards for single-case designs (Handbook, n.d.).

#### Inclusion Criteria:

1) Participants must be between 5 and 10 years of age, 2) able to ingest 1 Lightning Stik^TM^/day), 3) the children’s parents need to have English language competence sufficient to understand study materials to answer questionnaires, 4) the children need to have English language competence sufficient to understand and answer the questions asked in the child-related measure, 5) participants need to have at least mild-to-moderate levels of emotional dysregulation and irritability following the Affective-Reactivity-Index (ARI), represented by a total score of 3 or higher.

#### Exclusion criteria:

1) Taking psychotropic medications (e.g., Ritalin) in the prior 4 weeks before for the trial period. 2) presence of any serious medical or psychiatric condition that might require hospitalization, 3) any known allergies to the ingredients of the Lightning Stiks^TM^.

#### Product and dosing:

The product being tested is called EMP Lightning Stiks^TM^ which come as powdered straws that use Direct-to-Mouth technology, which means all the participants have to do is put the powder directly in their mouth and let it dissolve (rather than swallowing it). The Lightning Stiks^TM^ consist of a blend of 36 ingredients, including vitamins, minerals and amino acids. The advantage of this mode of delivery is that the doses of the ingredients are much lower than those found in capsules and well below the RDA - as such, no ingredient is given in a medicinal dose. Because the doses are all below RDA, safety concerns for any of the proposed ingredients are unlikely. Further, all doses are well below the Tolerable Upper Intake Limit (UL), which is considered the highest amount of daily nutrient intake that is not likely to cause any adverse effects (Nutritional Institutes of Health, n.d.). In addition, as the Lightning Stiks^TM^ are available in various flavours, such as Tropical Punch, Banana, and Sour Berry, they do not have the usual unpleasant smell or taste that multinutrients usually do, and no swallowing capsules is necessary, which is why they might be a convenient alternative treatment for children. In the proposed study, children will be able to choose which flavour they would like to try during the study. If the child would like to change the flavour of the Lightning Stiks^TM^ during an ON-phase they are welcome to do so.

Participants are asked to take one Lightning Stik^TM^ a day; however, as anecdotal reports have previously reported headaches in the first few days of taking the Lightning Stiks^TM^, participants are advised to start with only ½ a straw in this study for the first two days.

### Assessment Tools

An on-line survey created with Qualtrics will ask the participants and their parents to complete measures of emotion dysregulation, irritability, inattention, hyperactivity/impulsivity, diet, sleep, depression, anxiety and stress. The measures that are proposed in this study are standard measures used in clinical trials. In addition, the study coordinator will meet with the participants once a month in person at Te Puna Toiora to collect data on emotion dysregulation, mood, side effects, and interpersonal skills. If participants cannot be met in person, these meetings will be held via Zoom or other online platforms.

Primary outcome measures

*Clinician-administered:*

*The Clinician Affective Reactivity Index (Parent/Child Interview Guide) (CL-ARI):* This scale is a parent-report measure that assesses emotion regulation and irritability in children over the past week, looking at temper outbursts and irritable mood, and their frequency, severity, and duration. Further, it assesses whether the child’s irritable behaviour causes problems in the family, impairments in school, and assesses potential improvements over time. The CL-ARI has good internal consistency (*α* = .89) and adequate test-retest reliability (*ICC* = .67) (Haller et al., 2020). In the proposed study, the study coordinator will meet the parent and the child every four weeks to collect data on the CL-ARI in person.

*Clinical Global Impressions - Improvement Scale (CGI-I) (Guy, 1976):* The CGI-I is a clinician-administered scale that rates, based on all information obtained from all raters (parents, child and significant other) how much better/worse the participant is functioning since baseline across irritability/emotion dysregulation, mood, stress, energy, and sleep. Each item has seven responses, ranging from “very much improved” (1) to “very much worse” (7) and helps to provide a brief, overall assessment of the participant. The CGI-I is not used during baseline assessment. The CGI-I has demonstrated high external validity (*r* = 0.74) (Kadouri et al., 2007) and is widely used in clinical trials.

Secondary outcome measures

*Clinician-administered:*

*Clinical Global Impressions – Severity Scale (CGI-S) (Guy, 1976)* is a clinician-rated evaluation of symptom severity by means of the question: “Considering your total clinical experience with this particular population, how impaired is the patient at this time?” The item is scored on a 7-point Likert scale, ranging from 1 (normal, not impaired) to 7 (markedly impaired) (Busner, 2007). The CGI-S is commonly used in clinical trials and has shown to be sensitive to clinical change in diagnostically diverse populations. In the proposed study, it is administered during the baseline assessment and at the end of the study, giving an overall impression of the participants’ functioning before and after the intervention.

*Parent- or other-adult administered:*

*Affective Reactivity Index (ARI) (Parent version)* (Stringaris et al., 2012): The ARI is a concise, parent-rated scale assessing the child’s irritability. It incorporates seven items that ask about feelings of irritability during the past seven days (e.g., “My child is easily annoyed by others”, “My child gets angry frequently”, “Overall, irritability causes my child problems”). How true a statement is can be scored on a 0 (not true) to 2 (certainly true) Likert scale. It has been shown to have good internal consistency (*α* = .84 - .92) (Ezpeleta et al., 2020) and is considered an appropriate measure to assess irritability (Stringaris et al., 2012).

*Strengths and Difficulties Questionnaire (SDQ)* (Goodman, 1997): The Strength and Difficulties Questionnaire consists of 25 items across five subscales, measuring emotional symptoms, conduct problems, hyperactivity/inattention, peer relationship problems, and prosocial behavior in 4-17 year old children and adolescents (Stone, Otten, Engels, Vermulst, & Janssens, 2010). Each item is scored on a 3-point Likert scale (‘not true’, ‘somewhat true’, and ‘certainly true’) by the child’s parents. Scores between 0-13 are considered normal, whereas scores between 17-40 represent severe psychological symptoms.

The SDQ also looks at how severely the observed symptoms interfere with family relationships, friendships, learning in the classroom, and leisure activities. This ‘impact score’ is assessed by means of a 0 to 4 Likert scale (‘not at all’ to ‘a great deal’), with a score of 2 or greater representing problems in such areas (Gordon, Rucklidge, Blampied, & Johnstone, 2015). The SDQ is considered sufficiently internally consistent for the total difficulties scale (*α* = 0.78) as well as for the emotional problems subscale (*α* = 0.73). However, for the hyperactivity-inattention and prosocial behaviours its internal consistency is moderate (*α* = 0.69 and *α* = 0.63 respectively), and poor for peer and conduct problems (*α* = 0.57 and *α* = 0.47 respectively) (Bøe, Hysing, Skogen, & Breivik, 2016).

*Adapted Clinical Global Impressions Improvement Scale (CGI-I_Parent_) (Guy, 1976):* The original CGI-I was a clinician-administered scale that rates how much better/worse the participant is since participation in the trial across irritability/emotion dysregulation, mood, stress, sleep, and energy. Each item has seven responses, ranging from “very much improved” (1) to “very much worse” (7) and helps to provide a brief, overall assessment of the participant. In the proposed study, the CGI-I will also be adapted so that it represents the parent’s perception of change in their children’s behaviour (CGI_I_Parent_). In the proposed study, the CGI-I is not used during baseline assessment. The CGI-I has demonstrated high external validity (*r* = 0.74) (Kadouri et al., 2007) and is widely used in clinical trials.

*The Child Swanson, Nolan, and Pelham-IV Questionnaire (SNAP-IV) (Swanson, 1992; Swanson et al., 1983):* The child SNAP-IV assesses symptoms of ADHD and oppositional defiant disorder (ODD) in children every 4 weeks. It is a parent- and teacher-rating scale that has three subscales: inattention (item 1-9), hyperactivity-impulsivity (item 10-18), and symptoms of ODD (item 19-26), which are each scored on a 4-point Likert scale (‘not at all’, ‘just a little’, ‘quite a bit’, and ‘very much’). The SNAP-IV displays high internal consistency (*α* =.94) and sufficient interrater reliability and test-retest reliability (*r* =.43-.49) (Bussing et al., 2008). In the proposed study, the SNAP-IV will be emailed to another significant adult in the children’s life, for example aunties/uncles, grandparents, or teachers (in addition to the parent) at baseline and during the last month where participants take the nutrients.

*The Children’s Sleep Habits Questionnaire (CSHQ) (Owens et al., 2000):* The CSHQ is a parent-rated measure that assesses sleep in school-aged children. It consists of eight subscales that are both scored individually and make up a total score, identifying sleep problems. In the proposed study, only the total score will be reported, with a score of 41 (larger = more severe) representing the clinical cut-off score for sleep problems. Larger scores indicate more severe sleeping problems. The CSHQ has sufficient internal consistency (*α* = 0.68-0.78), adequate test-retest reliability (*r* = 0.62-0.79), a sensitivity of 0.80, and a specificity of 0.72 (Owens et al., 2000).

*Eating Behaviour Questionnaire (Baker et al., 2003)*: By means of a dietary questionnaire the parent’s and child’s general diet and eating behaviour is assessed. In particular, parents fill out various questions representing their and their child’s diet and eating pattern, including how many servings of fruit and vegetables they eat per day, how much food they eat per meal, how regular they eat, and how frequently they eat processed foods and fast-food. Lastly, participants are asked to rate their diet on a scale of 1 (not very healthy) to 7 (very healthy). The total score ranges from 9-47, and a higher score represents a healthier diet. Parents answer the dietary questionnaire during the first and the last study phase.

*Abbreviated Acceptability Rating Profile (AARP) (Tarnowski & Simonian, 1992).* At the end of the trial, parents will be asked whether they perceive the Lightning Stiks^TM^ as a helpful and acceptable treatment to reduce the children’s irritability and emotional dysregulation by means of the AARP. The AARP is a parent-administered, 8 item rating-scale, with each item being scored on 6-point Likert scale (1 = ’strongly disagree’ to 6 = ‘strongly agree’) asking about the child’s treatment acceptability, likeability, effectiveness, and helpfulness of the intervention. The AARP has shown to have excellent internal consistency (*α =* .95-.98) and adequate discriminant validity (Tarnowski & Simonian, 1992).

*The Depression Anxiety and Stress Scale-21 (DASS-21):* The DASS-21 is the shorter, 21-item of the original 42-item DASS-42 (Lovibond and Lovibond, 1995). It is a publicly available questionnaire, which assesses an individual’s symptom severity associated with depression, anxiety and stress using a 0 – 3 Likert scale. Cut-offs have been provided to indicate normal, mild, moderate, severe, or extremely severe problems; anything below 10 (for depression), 8 (for anxiety) and 15 (for stress) is considered within the normal to mild range. The scale has excellent reliability (*ICC* = .93) as well as a sufficient construct validity (Henry & Crawford, 2005). In the proposed study, it is completed by the parents to measure their own’s wellbeing and stress every four weeks.

*The Side-Effect Checklist (revised) (ASEC) (Uher et al., 2009):* The ASEC will assess side effects, safety and adverse events of the intervention. The ASEC has been adapted for the purposes of this study to remove references to anti-depressants. In addition, the item regarding sexual dysfunction is removed since it is not applicable for children, and the potential adverse event ‘rash’ was added because this has been a common side effect in past studies. Side effects will be assessed every two weeks, and any concerning adverse events will be discussed with the study physician and the co-investigators. In the case of a serious adverse event, the investigators will consider whether termination of the study is necessary.

*Parent- and child administered*

*The Measure Yourself Medical Outcome Profile (The MYMOP)* (Paterson, 1996): Once a month, the research coordinator will meet with the parent and the child to collect information in person regarding the child’s symptoms, including hyperactivity, attention, impulsivity, emotion regulation, irritability, mood, and sleep by means of the MYMOP. Each symptom can be scored on a scale from 0 to 5, with a score of 0 representing no problems, whereas a score of 5 indicates that there are lots of problems. The clinician will also ask questions regarding intervention compliance (on behalf of the children, the parents will report how easy it was to remember and to take the Lightning Stiks^TM^ on a 3-point Likert scale; ‘never difficult’, ‘sometimes difficult’, and ‘always difficult’), as well as potential side-effects, which can be scored on a 5-point Likert scale ranging from major problems to no problems. The MYMOP has demonstrated adequate internal consistency and validity (Hourigan, Clarke, Whitehouse, & Challinor, 2014).

## Statistical Analysis

***Exploratory analysis***

Before the data will be further analysed, all data sets will be cleaned. The demographic data of the participants will be presented in a table, describing gender, age, education, and medical history of each participant. For each variable at each time point group data in the form of the range, median, mean, and standard deviation will be tabulated, frequency distributions plotted and examined, and missing or anomalous data points investigated. Missing data will be handled following the ‘last observation carried forward’ method. Correlations between measures within and across time points will be examined to assess consistency (within time) and predictability (across time and measures) of responding.

***Ideographic analysis***

Graphical procedures such as spaghetti plots will be used to examine consistency of individual response over time within the study phases (where there are multiple time points). Modified Brinley Plots (Blampied, 2017) will examine the consistency of patterns of response to treatment and replications of such patterns over study phases. Sigma Plot will be used to examine individual change across time in the context of all other participants’ scores at that time, along with measures of means and SDs. In addition, effect sizes (ES), namely Cohen’s *d,* the 95%CI on *d*, and the Common Language ES (CLES; Lakens, 2013) will be reported. This gives the probability, expressed as a %, that a randomly selected participant will have an improved score at the second measurement point relative to the first measurement. Lakens (2013) software will be used to calculate the CLES and ESCI (Cumming, 2012) to compute *d* and the CI.

The ideographic analysis will also use single-case visual analysis to examine the extent to which there is replication of treatment effects across individuals and replicated phases. Note that the design means that the initial phases for the two groups will permit detection of any initial treatment effect for those in group 1 period 2 relative to those in group 2 period 2. The respective transitions into respectively group 1 period 3 and group 2 period 3 will permit observation of (a) any loss of therapy effect for group 1 changes in therapeutic direction (going ON to OFF) and any changes in the therapeutic direction for group 2 (going OFF to ON). Subsequent phase changes will permit observation of any replication of these effects, with the possibility of accumulation of therapy effects across phases as observed by Gordon et al. (2015).

Consistency analysis will examine consistency of individual response across primary outcome measures, i.e., to establish if an individual with a clinical level of response on any one DV will also have displayed similar positive responses on other DVs. Also, at the end of each treatment phase, participants may be classified using primary outcome measures as ‘Responders’ and ‘Non-responders’.

***Nomothetic analysis***

The ideographic analysis described above will be complemented by nomothetic analyses, which will involve hierarchical linear modeling. Baseline demographic variables will be examined using multiple regression and discriminant function analysis to determine if there are predictors of response to treatment.

***Moderator analysis***

Depending on the characteristics of the sample that will be recruited, demographic and other variables can be explored to identify moderators and other predictors of the treatment response. Other than using the software of Lakens (2103) and Cumming (2102), analyses will be carried out using the Statistical Package for Social Science (SPSS), Excel, Jamovi, and Sigma Plot.

## Anticipated costs

*Participant reimbursement*: The costs of this study are being funded by the University of Canterbury (funds provided to PhD students) and the University of Canterbury Foundation. At the beginning of the study, participants will need to meet with the study coordinator at the Mental Health and Nutrition Research Lab, Te Puna Toiora, to sign the informed consent form and to discuss any questions or concerns they might have with regards to the study. If the participants cannot be met in person, the meeting will take place online (e.g. via Zoom). Further, they will need to meet the study coordinator (every four weeks to answer parent- and child-related questionnaires as well as to provide information on compliance and potential side-effects of the intervention. To cover their driving costs to university, the participants are compensated with a $10 petrol voucher every time they meet the study coordinator at the university.

If the participants do not live in central Christchurch, or in case of New Zealand moving up to alert level 2, 3, or 4, the Lightning Stiks will need to be couriered to the participants as a meeting in person will not be possible. As a consequence, a mail cost of $30 per participant is necessary, as each participant will receive five packages containing the Lightning StiksTM, costing $6 each.

## Resources

The product we are testing is provided free of charge by the manufacturer (True Hope). They do not provide any financial support and are not involved in the study in any other way.

## Timetable

| **Date** | **Event** |
| --- | --- |
| February 2022 | Recruitment starts |
| February 2023 | Recruitment ends |
| August 2023 | Data analysis starts |

**The timeline might change based on how quickly approval from the Human and Disability Ethics Committee is granted. The earliest point in time recruitment might start would be September 2021.

## Ethics

The overall safety of all involved in of the outmost importance. The following safety protocol outlines what safety measures have been put in place to ensure safety of researchers and participants at the University.

## Safety protocol related to visits at the University Lab:

The University of Canterbury has very clear Health and Safety procedures for all visitors. We have a dedicated Health and Safety Team at the University: <https://www.canterbury.ac.nz/about/health-and-safety/>. The Mental Health and Nutrition Lab, Te Puna Toiora, is situated within the extensive facilities of the School of Psychology, Speech and Hearing, closely adjacent to clinics for audiology, clinical psychology, and speech therapy. All students and staff are trained to deal with emergency situations, such as evacuations, fire, and earthquakes. The Director of the Te Puna Toiora Mental Health and Nutrition Lab is a First Aid Certificate holder and we are equipped with a First Aid Kit. All short-term visitors are briefed as to the Emergency evacuation procedures. All staff and students who work with individuals under the age of 18 obtain a police check. The Director of the lab (Julia Rucklidge), Prof. Neville Blampied, a research investigator involved in this study, and one of the current PhD students are registered psychologists such that in the event of any concerns for the wellbeing of participants, we have on hand trained professionals capable of dealing with such circumstances that may arise. In addition, within 20 meters of the lab there are a further 6 university staff who are registered psychologists available for consultation should the need arise.

Participant safety is the most important concern. Every potential participant will be screened to ensure that there are no known physical/mental or metabolic (e.g. hemochromatosis – an inability to metabolise iron; Wilson’s disease – an inability to metabolize copper) conditions that may preclude participation. Safety of the intervention will be assessed once a month during the study period via a survey examining side effects and adverse events. When a serious adverse event is reported, it will be discussed with the study physician who will determine the appropriate course of action. In the case of worsening psychiatric symptoms or suicidal ideation with plan and intent, contact will be made with appropriate mental health services.

Research participants will be informed if any new information regarding the products' safety is brought to our attention during the trial. If we find that a child’s symptoms have increased to a clinically significant degree or that the product is causing any harm, we may discuss with the participant and their family the possibility of withdrawing them from the trial or may decide that they should discontinue their participation in the trial. Participants may be referred to their General Practitioner until the condition associated with an adverse event has resolved or until it is stable. All adverse events will be documented. Serious adverse events would include events that result in hospitalisation, life-threatening disability or death.

If a participant, for any reason, requires treatment with certain therapeutic agents (i.e. antibiotics), we will note what they are taking and for how long. If a protocol exclusion violation has occurred (i.e., participant requires psychiatric medications), the participant’s involvement will be discontinued. If any participant is discontinued from the trial or decides to withdraw, we will carry out follow-ups to confirm the well-being of participants.

Potential side effects

As mentioned previously, anecdotal reports have reported that as a consequence of taking the Lightning Stiks, individuals may suffered from headaches in the first few days of taking them. However, this could potentially be minimized by taking only half a Stik initially, and increasing the dose over 2 days. Further, the doses of the ingredients are much lower than those found in capsules and well below the RDA - as such, no ingredient is given in a medicinal dose. Because the doses are all below RDA, safety issues for any of the proposed ingredients are very unlikely. Further, all doses are well below the Tolerable Upper Intake Limit (UL), which is considered the highest amount of daily nutrient intake that is not likely to cause any adverse effects (Nutritional Institutes of Health, n.d.). This low risk of adverse effects was supported by the findings of the STAR trial (not published), showing that only a small number of side effects were present in the study (drowsiness and reduced appetite), and these were temporary and disappeared during the duration of the trial.

Further, in an RCT conducted at the University of Canterbury with adults (Rucklidge et al, 2014), the only recurring side effect of the formula was the aforementioned transitory gastrointestinal difficulties (loose stool, nausea or vomiting, if the product was taken on an empty stomach, contrary to recommendation) although this side effect did not occur more frequently in the active group as compared with the placebo group. It is important to note that this side effect typically lasts only a few days and can be addressed by staying hydrated. In addition, there have been 34 studies to date on the EMP micronutrient formula showing that no serious side effects or other effects have been noted. A recent safety study has been published documenting its safety and tolerability: Rucklidge, J., Eggleston, M. J., Ealam, B., Beaglehole, B., & Mulder, R. T. (2019). An observational preliminary study on the safety of long-term consumption of micronutrients for the treatment of psychiatric symptoms. *The Journal of Alternative and Complementary Medicine*, *25*(6), 613-622.

Cultural sensitivity

We have consulted with the Māori Research Advisory Group at the University of Canterbury regarding this type of research and the study has received approval to conduct nutritional research with Māori. The Ngai Tahu Consultation and Engagement Group (NTCEG) was consulted on 22/07/2021 by means of the Māori Consultation form and they have approved the study process on 30/07/2021. No issues were identified, and further consultation with Māori is not required. The group is available for further consultation throughout the project.

Within Te Puna Toiora and in the proposed research project, providing a holistic approach to mental health is the highest priority, aiming to begin to address the inequity in health outcomes between Māori and non-Māori by being culturally responsive. In the proposed research project, members of all ethnicities are invited to participate, with an emphasis on increasing Māori participation. Specifically, Māori service providers will be approached in order to reach Māori whānau. Further, measures will be put in place to ensure that Māori customs and traditions will be respected, such as having a cultural advisor available to provide extra support if needed. We also consulted the cultural advisor on the design of our previous projects and the design of this one is modelled on that of others that have received her input. In addition, this research aligns with the Māori health model Te Whare Tapa Whā as the role of kai in wellbeing has long been understood by Māori. Focusing on nutrition to improve mental health takes steps in upholding mātaranga Māori. Indeed, three of the four cornerstones of Te Whare Tapa Whā are represented within this research; taha tinana (physical health), taha hinengaro (mental health), and taha whānau (family health). Further, over the last year, the Mental Health and Nutrition Research lab, Te Puna Toiora, has been focusing on increasing Te Reo used within the lab, which is reflected in being gifted a Māori name, translating words within the lab into Māori, and having bilingual business cards. We will ensure that Te Reo is used within the information sheet as well to ensure that the research is perceived as welcoming for Māori. Finally, participants have the option to discuss with the research team any queries or worries that they may have at any time. These options will be clearly communicated via the information sheet, which individuals are advised to read before commencing participation.

## Research site

Te Puna Toiora: Mental Health and Nutrition Laboratory; Room 465

School of Psychology, Speech and Hearing

University of Canterbury

Private Bag 4800

Christchurch 8140

New Zealand

## Investigators

*Nurina Katta:* Coordinator/Investigator. Nurina Katta will be the participant’s primary person to contact in case of questions or concerns. The proposed study is part of a PhD in Psychology.

*Prof. Julia Rucklidge*: Primary Investigator/supervisor. Prof. Rucklidge will oversee the running of the study and provide support and supervision to researchers on the project, being available at all times should any difficulties arise.

*Prof. Neville Blampied:*  Co-Investigator. Prof. Blampied will provide support and supervision to researchers on the project with specific expertise in case study design.

*Prof. Matt Eggleston*: consulting psychiatrist to the team.

## Risk Management

As the principal investigator and as someone located on site for data collection, the responsibility for risk management (such as managing psychological symptoms) of the project will be undertaken by Prof. Rucklidge (registered clinical psychologist). Prof. Rucklidge will be consulted on all aspects of the project and will therefore be aware of any foreseeable risks which can then be reviewed and cleared with the study physician Dr. Matt Eggleston. Any serious adverse effects will be reported to the ethics committees (Human Disability Research Ethics Committee and Human Ethics Committee at University of Canterbury), and the trial will be terminated if serious adverse effects known to be caused by the nutrients occur.

## Data ownership

All data associated with the study and all reports resulting from the same will be owned by the authors.

## Conflicts of interest

There is no conflict of interest in the proposed study. The supplements used in this trial will be donated by the manufacturers. Study investigators have no financial affiliations with the manufacturer of the product.

# References

Advokat, C. (2010). What are the cognitive effects of stimulant medications? Emphasis on adults with attention-deficit/hyperactivity disorder (ADHD). *Neuroscience & Biobehavioral Reviews*, 34(8), 1256-1266. https://doi.org/10.1016/j.neubiorev.2010.03.006

American Psychiatric Association. (2013). Diagnostic and statistical manual of mental disorders (5th ed.). https://doi.org/10.1176/appi.books.9780890425596

Andersen, S. L. (2005). Stimulants and the developing brain. Trends in pharmacological sciences, 26(5), 237-243. https://doi.org/10.1016/j.tips.2005.03.009

Bailey, R. L., West Jr., K. P., & Black, R. E. (2015). The epidemiology of global multinutrient deficiencies. *Annals of Nutrition and Metabolism*, *66*(2), 22-33. https://doi.org/10.1159/000371618

Baker, C. W., Little, T. D., & Brownell, K. D. (2003). Predicting adolescent eating and activity behaviors: The role of social norms and personal agency. *Health Psychology*, *22*(2), 189. https://doi.org/10.1037/0278-6133.22.2.189

Barbaresi, W. J., Katusic, S. K., Colligan, R. C., Weaver, A. L., Leibson, C. L., & Jacobsen, S. J. (2014). Long-term stimulant medication treatment of attention-deficit/hyperactivity disorder: Results from a population-based study. Journal of Developmental & Behavioral Pediatrics, 35(7), 448-457. https://doi: 10.1097/DBP.0000000000000099

Barkley, R. A. (2006). *Attention-Deficit/Hyperactivity Disorder.* Guilford Publications.

Baumgaertel, A. (1999). Alternative and controversial treatments for attention-deficit/hyperactivity disorder. *Pediatric Clinics of North America*, 46(5), 977-992. https://doi.org/10.1016/S0031-3955(05)70167-X

Beauchaine, T. P. (2015). Future directions in emotion dysregulation and youth psychopathology. *Journal of Clinical Child & Adolescent Psychology*, *44*(5), 875-896. https://doi.org/10.1080/15374416.2015.1038827

Beauregard, M., Lévesque, J., & Bourgouin, P. (2001). Neural correlates of conscious self-regulation of emotion. *Journal of Neuroscience*, *21*(18), 1-6. https://doi.org/10.1523/JNEUROSCI.21-18-j0001.2001

Biederman, J., Spencer, T., & Wilens, T. (2004). Evidence-based pharmacotherapy for attention-deficit hyperactivity disorder. *International Journal of Neuropsychopharmacology*, *7*(1), 77-97. https://doi.org/10.1017/S1461145703003973

Blampied, N. M. (2017). Analyzing therapeutic change using modified Brinley plots: History, construction, and interpretation. *Behavior Therapy*, *48*(1), 115-127. https://doi.org/10.1016/j.beth.2016.09.002

Bøe T, Hysing M, Skogen JC, Breivik K (2016). The strengths and difficulties questionnaire (SDQ): Factor structure and gender equivalence in norwegian adolescents*. PLoS ONE* *11*(5), 131-142. https://doi.org/10.1371/journal.pone.0152202

Broman, J.-E., Smedje, H., Mallon, L., & Hetta, J. (2008). The minimal insomnia symptom scale (MISS). *Upsala Journal of Medical Sciences*, *113*(2), 131-142. https://doi.org/10.3109/2000-1967-221

Brotman, M. A., Kircanski, K., & Leibenluft E. (2017). Irritability in children and adolescents. *Annual Review of Clinical Psychology. 13*, 317–341. https://doi.org/10.1146/annurev-clinpsy-032816-044941

Brotman, M. A., Schmajuk, M., Rich, B. A., Dickstein, D. P., Guyer, A. E., Costello, E. J., Egger, H. L., Angold, A., Pine, D. S. & Leibenluft, E. (2006). Prevalence, clinical correlates, and longitudinal course of severe mood dysregulation in children. *Biological Psychiatry*, *60*, 991-997. http://doi.prg/10.1016/j.biopsych.2006.08.042

Bussing, R., Fernandez, M., Harwood, M., Hou, W., Garvan, C. W., Eyberg, S. M., & Swanson, J. M. (2008). Parent and teacher SNAP-IV ratings of attention deficit hyperactivity disorder symptoms: Psychometric properties and normative ratings from a school district sample. *Assessment*, *15*(3), 317-328. https://doi.org/10.1177/1073191107313888

Carlson, S. M., & Wang, T. S. (2007). Inhibitory control and emotion regulation in preschool children. *Cognitive Development*, *22*(4), 489-510. https://doi.org/10.1016/j.cogdev.2007.08.002

Coccaro, E. F., Lee, R. J. & Kavoussi, R. J. (2009). A double-blind, randomized, placebo-controlled trial of fluoxetine in patients with intermittent explosive disorder*. Journal of Clinical Psychiatry,* *70*(65), 3-62

Cumming, G. (2013). *Understanding the new statistics: Effect sizes, confidence intervals, and meta-analysis*. Routledge. https://books.google.co.nz/books?hl=en&lr=&id=1W6laNc7Xt8C&oi=fnd&pg=PR1&dq=Cumming,+G.+(2102).+Understanding+the+New+Statistics.+&ots=PwFTWFgZ1R&sig=onOycx_mXJBEPH0du5dGe7FnNm4&redir_esc=y#v=onepage&q&f=false

Currie, J., Stabile, M., & Jones, L. (2014). Do stimulant medications improve educational and behavioral outcomes for children with ADHD? *Journal of Health Economics*, *37*, 58-69. https://doi.org/10.1016/j.jhealeco.2014.05.002

Daley, D., Van der Oord, S., Ferrin, M., Danckaerts, M., Doepfner, M., Cortese, S., Sonuga-Barke E. J. S., & European ADHD Guidelines Group. (2014). Behavioral interventions in attention-deficit/hyperactivity disorder: A meta-analysis of randomized controlled trials across multiple outcome domains. *Journal of the American Academy of Child & Adolescent Psychiatry*, *53*(8), 835-847. https://doi.org/10.1016/j.jaac.2014.05.013

Davidovitch, M., Koren, G., Fund, N., Shrem, M., & Porath, A. (2017). Challenges in defining the rates of ADHD diagnosis and treatment: Trends over the last decade. *BMC Pediatrics*, *17*(1), 1-9. http://doi.org/10.1186/s12887-017-0971-0

Denham, S. A., Blair, K. A., DeMulder, E., Levitas, J., Sawyer, K., Auerbach–Major, S., & Queenan, P. (2003). Preschool emotional competence: Pathway to social competence? *Child Development*, *74*(1), 238-256. https://doi.org/10.1111/1467-8624.00533

DuPaul, G. J., Power, T. J., Anastopoulos, A. D., & Reid, R. (2016). *ADHD rating scale? 5 for children and adolescents: Checklists, norms, and clinical interpretation.* Guilford Publications.

Eisenberg, N., Fabes, R. A., Bernzweig, J., Karbon, M., Poulin, R., & Hanish, L. (1993). The relations of emotionality and regulation to preschoolers' social skills and sociometric status. *Child Development*, *64*(5), 1418-1438. https://doi.org/10.1111/j.1467-8624.1993.tb02961.x

Ezpeleta, L., Penelo, E., de la Osa, N., Navarro, J. B., & Trepat, E. (2020). How the Affective Reactivity Index (ARI) works for teachers as informants. *Journal of Affective Disorders*, *261*, 40-48. http://doi.org/10.1016/j.jad.2019.09.080.

Food and Agriculture Organization of the United Nations (FAO) (2017). The future of food and agriculture: Trends and challenges. Rome. http://www.fao.org/3/i6583e/i6583e.pdf

Garnefski, N., Rieffe, C., Jellesma, F., Terwogt, M. M., & Kraaij, V. (2007). Cognitive emotion regulation strategies and emotional problems in 9–11-year-old children. *European child & Adolescent Psychiatry*, *16*(1), 1-9. https://link.springer.com/content/pdf/10.1007/s00787-006-0562-3.pdf

Geller, B., Zimerman, B., Williams, M., Bolhofner, K., Craney, J. L., DelBello, M. P., & Soutullo, C. (2001). Reliability of the Washington University in St. Louis Kiddie Schedule for Affective Disorders and Schizophrenia (WASH-U-KSADS) mania and rapid cycling sections. *Journal of the American Academy of Child & Adolescent Psychiatry*, *40*(4), 450-455. https://doi.org/10.1097/00004583-200104000-00014

Gioia, G. A., Isquith, P. K., Guy, S. C., & Kenworthy, L. (2013). *BRIEF – Behavior Rating Inventory of executive function*. PAR.

Goodman, R. (1997). The strengths and difficulties questionnaire: A research note. *Journal of Child Psychology and Psychiatry*, *38*(5), 581-586. https://doi.org/10.1111/j.1469-7610.1997.tb01545.x

Goodman, R. (2001). Psychometric properties of the strengths and difficulties questionnaire. *Journal of the American Academy of Child & Adolescent Psychiatry*, *40*(11), 1337-1345. https://doi.org/10.1097/00004583-200111000-00015

Gordon, H. A., Rucklidge, J. J., Blampied, N. M., & Johnstone, J. M. (2015). Clinically significant symptom reduction in children with attention-deficit/hyperactivity disorder treated with micronutrients: An open-label reversal design study. *Journal of Child and Adolescent Psychopharmacology*, *25*(10), 783-798. https://doi.org/10.1089/cap.2015.0105

Guy, W. (1976). *ECDEU assessment manual for psychopharmacology*. US Department of Health, Education, and Welfare. https://books.google.co.nz/books?hl=en&lr=&id=s81_xfFZV2EC&oi=fnd&pg=PA6&dq=Guy+US+Department+of+Health,+Education+and+Welfare%3B+1976.+p.+534%E2%80%937.+++&ots=-p6TvOiy6f&sig=i2by9lqPEzFoFrgGaFkip_fbiPU&redir_esc=y#v=onepage&q&f=false

Haller, S. P., Kircanski, K., Stringaris, A., Clayton, M., Bui, H., Agorsor, C., Cardenas, S. I., Towbin, K. E., Pine, D. S., & Leibenluft, E. (2020). The clinician affective reactivity index: Validity and reliability of a clinician-rated assessment of irritability. *Behavior Therapy,* *51*(2), 283-293. https://doi.org/10.1016/j.beth.2019.10.005

Hersen, M. & Barlow, D. (1976). *Single-case experimental designs: Strategies for studying behaviour change*. Pergamon Press.

Hourigan, P., Clarke, A., Whitehouse, S. L., & Challinor, H. M. (2014). Measure your own medical outcome profile (MYMOP): Validation of a patient generated outcome questionnaire by comparison to the Roland Morris disability questionnaire (RMDQ). *The Spine Journal,* *14*(11), 1S–183S. https://doi.org/10.1016/j.spinee.2014.08.136

Huang, C. J., Webb, H. E., Zourdos, M. C., & Acevedo, E. O. (2013). Cardiovascular reactivity, stress, and physical activity. *Frontiers in Physiology*, *4*(314), 1-13. https://doi.org/10.3389/fphys.2013.00314

Jacka, F. N., O’Neil, A., Opie, R., Itsiopoulos, C., Cotton, S., Mohebbi, M., Castle, D., Dash, S., Chatteron, M. L., Brazionis, L., Dean, O., hodge, A. M. & Berk, M. (2017). A randomised controlled trial of dietary improvement for adults with major depression (the ‘SMILES’trial). *BMC medicine*, *15*(1), 1-13. https://doi.org/10.1186/s12916-017-0791-y

Kadouri, A., Corruble, E., & Falissard, B. (2007). Improved Clinical Global Impression Scale [Database record]. *APA PsycTests.* https://doi.org/10.1037/t68608-000

Kamphaus, R. W. (1987). Conceptual and psychometric issues in the assessment of adaptive behavior. *The Journal of Special Education*, *21*(1), 27-35. https://doi.org/10.1177/002246698702100107

Kazdin AE. 2010. Problem-solving skills training and parent management training for oppositional defiant disorder and conduct disorder. In J. Weisz & A. Kazdin (Eds.), *Evidence-based psychotherapies for children and adolescents* (2nd ed.) (pp. 211–26). Guilford Press. Kessler, R. C., Adler, L., Barkley, R., Biederman, J., Conners, C. K., Demler, O., Faraone, S. V., Greenhill, L. L., Howes, M. J., & Secnik, K. (2006). The prevalence and correlates of adult ADHD in the United States: Results from the national comorbidity survey replication. *American Journal of Psychiatry*, *163*(4), 716-723. https://ajp.psychiatryonline.org/doi/pdf/10.1176/ajp.2006.163.4.716

Kopp, C. B. (1982). Antecedents of self-regulation: A developmental perspective. *Developmental Psychology*, *18*(2), 199-214. https://doi.org/10.1037/0012-1649.18.2.199

Lakens, D. (2013). Calculating and reporting effect sizes to facilitate cumulative science: A practical primer for t-tests and ANOVAs. *Frontiers in Psychology*, *4*, 863. http://doi.org/ 10.3389/fpsyg.2013.00863

Mertz, W. (1994). A balanced approach to nutrition for health: The need for biologically essential minerals and vitamins. *Journal of the American Dietetic Association*, *94*(11), 1259-1262. https://doi.org/10.1016/0002-8223(94)92456-2

Mick, E., Spencer, T., Wozniak, J., & Biederman, J. (2005). Heterogeneity of irritability in attention-deficit/hyperactivity disorder subjects with and without mood disorders. *Biological Psychiatry*, *58*(7), 576-582. https://doi.org/10.1016/j.biopsych.2005.05.037

Miller, B. F., Petterson, S., Burke, B. T., Phillips Jr, R. L., & Green, L. A. (2014). Proximity of providers: Colocating behavioral health and primary care and the prospects for an integrated workforce. *American Psychologist*, *69*(4), 443. http://doi.org/10.1037/a0036093

Moffitt, T. E., Arseneault, L., Belsky, D., Dickson, N., Hancox, R. J., Harrington, H., Houts, R., Poulton, R., Roberts, B. W., Ross, S., Sears, M., Thomson, W. M. & Caspi, A. (2011). A gradient of childhood self-control predicts health, wealth, and public safety. *Proceedings of the National Academy of Sciences*, *108*(7), 2693-2698. https://doi.org/10.1073/pnas.1010076108

Molina, B. S., Hinshaw, S. P., Swanson, J. M., Arnold, L. E., Vitiello, B., Jensen, P. S., Epstein, J. N., Hoza, B., Hechtman, L., & Abikoff, H. B. (2009). The MTA at 8 years: Prospective follow-up of children treated for combined-type ADHD in a multisite study. *Journal of the American Academy of Child & Adolescent Psychiatry*, *48*(5), 484-500. https://doi.org/10.1097/CHI.0b013e31819c23d0

Myers, S. B., Sweeney, A. C., Popick, V., Wesley, K., Bordfeld, A., & Fingerhut, R. (2012). Self-care practices and perceived stress levels among psychology graduate students. *Training and Education in Professional Psychology*, *6*(1), 55-66. https://doi.org/10.1037/a0026534

Nutritional Institutes of Health (n.d.). *Nutrient Recommendations: Dietary Reference Intakes (DRI).* https://ods.od.nih.gov/HealthInformation/Dietary_Reference_Intakes.aspx

Ochsner, K. N., Bunge, S. A., Gross, J. J., & Gabrieli, J. D. (2002). Rethinking feelings: An FMRI study of the cognitive regulation of emotion. *Journal of Cognitive Neuroscience*, *14*(8), 1215-1229. https://doi.org/10.1162/089892902760807212

Owens, J. A., Spirito, A., & McGuinn, M. (2000). The Children's Sleep Habits Questionnaire (CSHQ): Psychometric properties of a survey instrument for school-aged children. *Sleep-New York-*, *23*(8), 1043-1052. https://www.researchgate.net/profile/Anthony-Spirito/publication/12184585_The_Children%27s_Sleep_Habits_Questionnaire_CSHQ_Psychometric_properties_of_a_survey_instrument_for_school-aged_Children/links/541ffb3b0cf2218008d42a33/The-Childrens-Sleep-Habits-Questionnaire-CSHQ-Psychometric-properties-of-a-survey-instrument-for-school-aged-Children.pdf

Polaha, J., Dalton 3rd, W. T., & Lancaster, B. M. (2008). Parental report of medication acceptance among youth: Implications for everyday practice. *Southern Medical Journal*, *101*(11), 1106-1112. https://doi.org/10.1097/smj.0b013e318180645d

Parletta, N., Zarnowiecki, D., Cho, J., Wilson, A., Bogomolova, S., Villani, A., Itsiopoulos, C., Niyonsenga, T., Blunden, S., Meyer, B., Segal, S., Baune, B. T. & O'Dea, A. (2018). A Mediterranean-style dietary intervention supplemented with fish oil improves diet quality and mental health in people with depression: A randomised controlled trial (HELFIMED). *Journal of the Australasian College of Nutritional and Environmental Medicine*, *37*(1), 6-18. https://doi/10.3316/informit.534963995829643

Paterson, C. (1996). Measuring outcomes in primary care: A patient generated measure, MYMOP, compared with the SF-36 health survey. *BMJ*, *312*(7037), 1016-1020. doi: https://doi.org/10.1136/bmj.312.7037.1016

Posner, J., Kass, E., & Hulvershorn, L. (2014). Using stimulants to treat ADHD-related emotional lability. *Current Psychiatry Reports*, *16*(10), 478. http://doi.org/10.1007/s11920-014-0478-4

Psychol 22: 189–198

Pylypow, J., Quinn, D., Duncan, D., & Balbuena, L. (2020). A measure of emotional regulation and irritability in children and adolescents: The clinical evaluation of emotional regulation–9. *Journal of Attention Disorders*, *24*(14), 2002-2011. https://doi.org/10.1177/1087054717737162

Retz, W., Stieglitz, R.-D., Corbisiero, S., Retz-Junginger, P., & Rösler, M. (2012). Emotional dysregulation in adult ADHD: What is the empirical evidence? *Expert Review of Neurotherapeutics*, *12*(10), 1241-1251. https://doi.org/10.1586/ern.12.109

Rossignol, D. A., & Frye, R. E. (2012). Mitochondrial dysfunction in autism spectrum disorders: A systematic review and meta-analysis. *Molecular Psychiatry*, *17*(3), 290-314. http://doi.org/10.1038/mp.2010.136

Rucklidge, J. & Kaplan B. (2021). *The better brain: Overcome anxiety, combat depression, and reduce ADHD and stress with nutrition.* Houghton Mifflin Harcourt.

Rucklidge, J. J., & Blampied, N. M. (2011). Post-earthquake psychological functioning in adults with attention-deficit/hyperactivity disorder: Positive effects of micronutrients on resilience. *New Zealand Journal of Psychology*, *40*(4), 51. http://pamelaannezell.com/wp-content/uploads/sites/21/docs/Rucklidge_2011(3).pdf

Rucklidge, J. J., & Kaplan, B. J. (2013). Broad-spectrum multinutrient formulas for the treatment of psychiatric symptoms: A systematic review. *Expert Review of Neurotherapeutics*, *13*(1), 49-73. https://doi.org/10.1586/ern.12.143

Rucklidge, J. J., Andridge, R., Gorman, B., Blampied, N., Gordon, H., & Boggis, A. (2012). Shaken but unstirred? Effects of micronutrients on stress and trauma after an earthquake: RCT evidence comparing formulas and doses. *Human Psychopharmacology: Clinical and Experimental*, *27*(5), 440-454. https://doi.org/10.1002/hup.2246

Rucklidge, J. J., Eggleston, M. J., Ealam, B., Beaglehole, B., & Mulder, R. T. (2019). An observational preliminary study on the safety of long-term consumption of micronutrients for the treatment of psychiatric symptoms. *The Journal of Alternative and Complementary Medicine*, *25*(6), 613-622. https://doi.org/10.1089/acm.2018.0352

Rucklidge, J. J., Eggleston, M. J., Johnstone, J. M., Darling, K., & Frampton, C. M. (2018). Vitamin‐mineral treatment improves aggression and emotional regulation in children with ADHD: A fully blinded, randomized, placebo‐controlled trial. *Journal of Child Psychology and Psychiatry*, *59*(3), 232-246. https://doi.org/10.1111/jcpp.12817

Rucklidge, J. J., Frampton, C. M., Gorman, B., & Boggis, A. (2014). Vitamin–mineral treatment of attention-deficit hyperactivity disorder in adults: Double-blind randomised placebo-controlled trial. *The British Journal of Psychiatry*, *20*4(4), 306-315. doi:10.1192/bjp.bp.113.132126

Rucklidge, J., Taylor, M., & Whitehead, K. (2011). Effect of micronutrients on behavior and mood in adults with ADHD: Evidence from an 8-week open label trial with natural extension. *Journal of Attention Disorders*, *15*(1), 79-91. https://doi.org/10.1177/1087054709356173

Russell, V. A., Oades, R. D., Tannock, R., Killeen, P. R., Auerbach, J. G., Johansen, E. B., & Sagvolden, T. (2006). Response variability in attention-deficit/hyperactivity disorder: A neuronal and glial energetics hypothesis. *Behavioral and Brain Functions*, *2*(1), 1-25. http:// doi.org/10.1186/1744-9081-2-30

Saarni, C. (1984). An observational study of children's attempts to monitor their expressive behavior. *Child Development*, *55*, 1504-1513. https://doi.org/10.2307/1130020

Saarni, C., Campos, J.J., Camras, L.A. and Witherington, D. (2007). Emotional Development: Action, Communication, and Understanding. In W. Damon, R.M. Lerner and N. Eisenberg (Eds.), *Handbook of Child Psychology*, https://doi.org/10.1002/9780470147658.chpsy0305

Shaw, P., Stringaris, A., Nigg, J., & Leibenluft, E. (2014). Emotion dysregulation in attention deficit hyperactivity disorder. *American Journal of Psychiatry*, *171*(3), 276-293. https://doi.org/10.1176/appi.ajp.2013.13070966

Slutzke, W., Moffitt, T., Poulton, & Caspi, A. (2013). Undercontrolled temperament at age 3 predicts disordered gambling at age 32: A longitudinal study of a complete birth cohort. *Psychological Science* *23*(5), 510-516.

Sole, E. J., Rucklidge, J. J., & Blampied, N. M. (2017). Anxiety and stress in children following an earthquake: Clinically beneficial effects of treatment with micronutrients. *Journal of Child and Family Studies*, *26*(5), 1422-1431. https://link.springer.com/content/pdf/10.1007/s10826-016-0607-2.pdf

Stansbury, K., & Gunnar, M. R. (1994). Adrenocortical activity and emotion regulation. *Monographs of the Society for Research in Child Development*, *59*(2-3), 108-134. https://doi.org/10.1111/j.1540-5834.1994.tb01280.x

Stone, L. L., Otten, R., Engels, R. C., Vermulst, A. A., & Janssens, J. M. (2010). Psychometric properties of the parent and teacher versions of the strengths and difficulties questionnaire for 4-to 12-year-olds: A review. *Clinical Child and Family Psychology Review*, *13*(3), 254-274. https://link.springer.com/content/pdf/10.1007/s10567-010-0071-2.pdf

Stough, C., Scholey, A., Lloyd, J., Spong, J., Myers, S., & Downey, L. A. (2011). The effect of 90 day administration of a high dose vitamin B‐complex on work stress. *Human Psychopharmacology: Clinical and Experimental*, *26*(7), 470-476. https://doi.org/10.1002/hup.1229

Stringaris, A. (2011). Irritability in children and adolescents: A challenge for DSM-5. *European Child & Adolescent Psychiatry*, *20*, 61-66. http;//doi.org/10.1007/s00787-010-0150-4

Stringaris, A., Goodman, R., Ferdinando, S., Razdan, V., Muhrer, E., Leibenluft, E., & Brotman, M. A. (2012). The affective reactivity index: A concise irritability scale for clinical and research settings. *Journal of Child Psychology and Psychiatry*, *53*(11), 1109-1117. https://doi.org/10.1111/j.1469-7610.2012.02561.x

Swanson, J., Nolan, W., & Pelham, W. E. (1992). The SNAP-IV rating scale.

Tarnowski, K., & Simonian, S. (1992). Assessing treatment acceptance: The abbreviated acceptability rating scale. *Journal of Behavior Therapy and Experimental Psychiatry*, *23*(2), 101-106. https://doi.org/10.1016/0005-7916(92)90007-6

Terwogt, M. M., & Stegge, H. (1995). *Children’s understanding of the strategic control of negative emotions*. Springer.

Thomas, R., Mitchell, G. K., & Batstra, L. (2013). Attention-deficit/hyperactivity disorder: Are we helping or harming? *BMJ*, 347. https://doi.org/10.1136/bmj.f6172

Thompson, R. A. (1994). Emotion regulation: A theme in search of definition. *Monographs of the Society for Research in Child Development*, *59*(2-3), 25-52. https://doi.org/10.1111/j.1540-5834.1994.tb01276.x

Thompson, R. A. (2019). Emotion dysregulation: A theme in search of definition. *Development and Psychopathology*, *31*(3), 805-815. https://doi.org/10.1017/S0954579419000282

Towbin, K., Vidal-Ribas, P., Brotman, M. A., Pickles, A., Miller, K. V., Kaiser, A., Vitale, A., Engel, C., Overman, G. P., Davis, M., Lee, B., McNeil, C., Wheeler, W., Yokum, C. H., Haring, C. T., Roule, A., Wambach, C. G., Sharif-Askary, B., Pine, D. S., Leibenluft, E. & Stringaris, A. (2020). A double-blind randomized placebo-controlled trial of citalopram adjunctive to stimulant medication in youth with chronic severe irritability. *Journal of the American Academy of Child & Adolescent Psychiatry*, *59*(3), 350-361. https://doi.org/10.1016/j.jaac.2019.05.015

Uher, R., Farmer, A., Henigsberg, N., Rietschel, M., Mors, O., Maier, W., Kozel, D., Hauser, J., Souery, D., Placentino, A., Strohmaier, J., Perroud, N., Zobel., A., Rajewska-Rager, A., Dernovsek, M. Z., Larsen, E. R., Kalember, P., Giovannini, C., Barreto, M., McGuffin, P., & Aitchison, K. J. (2009). Adverse reactions to antidepressants. *The British Journal of Psychiatry*, *195*(3), 202-210. https://doi.org/10.1192/bjp.bp.108.061960

Wang, M., & Saudino, K. J. (2011). Emotion regulation and stress. *Journal of Adult Development*, *18*(2), 95-103. https://link.springer.com/content/pdf/10.1007/s10804-010-9114-7.pdf

Wong, J. G., Cheung, E. P., Chan, K. K., Ma, K. K., & Wa Tang, S. (2006). Web-based survey of depression, anxiety and stress in first-year tertiary education students in Hong Kong. *Australian & New Zealand Journal of Psychiatry*, *40*(9), 777-782. https://doi.org/10.1080/j.1440-1614.2006.01883.x

Young, L. T. (2007). Is bipolar disorder a mitochondrial disease? *Journal of Psychiatry & Neuroscience*, *32*(3), 160-161. https://www.ncbi.nlm.nih.gov/pmc/articles/PMC1863554/pdf/20070500s00001p160.pdf

Zeman, J., Cassano, M., Perry-Parrish, C., & Stegall, S. (2006). Emotion regulation in children and adolescents. *Journal of Developmental & Behavioral Pediatrics*, *27*(2), 155-168. file:///C:/Users/nmk25/AppData/Local/Temp/MicrosoftEdgeDownloads/04a829b3-0755-40ad-ba8b-becccbddfa96/Emotion_Regulation_in_Children_and_Adolescents.14%20(1).pdf

Zimmermann, L. K., & Stansbury, K. (2004). The influence of emotion regulation, level of shyness, and habituation on the neuroendocrine response of three-year-old children. *Psychoneuroendocrinology*, *29*(8), 973-98. https://doi.org/10.1016/j.psyneuen.2003.09.0

**Appendices**

## Appendix A: Lightning Stiks active intervention ingredient list


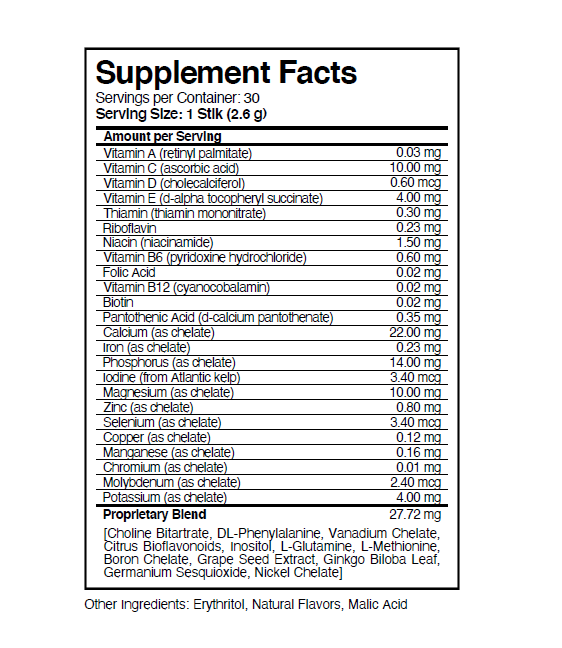


**Appendix B: Schedule of Events**

| **Measure** | **Group** | **Screening** | **Study phase 1** | **Study phase 2** | | **Study phase 3** | | **Study phase 4/5** |
| --- | --- | --- | --- | --- | --- | --- | --- | --- |
|  |  |  |  | **Week 2** | **Week 4** | **Week 2** | **Week 4** | **Week 2 – Week 12 (every 2-4 weeks)** |
| **CGI-I** | Group 1 |  |  | x | x | x | x | x |
|  | Group 2 |  |  | x | x | x | x | x |
| **CGI-S** | Group 1 |  | x |  |  |  |  | x |
|  | Group 2 |  | x |  |  |  |  | x |
| **SNAP-IV** | Group 1 |  | x |  | x |  | x | x |
|  | Group 2 |  | x |  | x |  | x | x |
| **CL-ARI** | Group 1 |  | x |  | x |  | x | x |
|  | Group 2 |  | x |  | x |  | x | x |
| **SDQ** | Group 1 |  | x | x | x | x | x | x |
|  | Group 2 |  | x | x | x | x | x | x |
| **ARI** | Group 1 | x | x | x | x | x | x | x |
|  | Group 2 | x | x | x | x | x | x | x |
| **MYMOP** | Group 1 |  | x |  | x |  | x | x |
|  | Group 2 |  | x |  | x |  | x | x |
| **DASS-21** | Group 1 |  | x |  | x |  | x | x |
|  | Group 2 |  | x |  | x |  | x | x |
| **CSHQ** | Group 1 |  | x | x | x | x | x | x |
|  | Group 2 |  | x | x | x | x | x | x |
| **EBQ** | Group 1 |  | x |  |  |  |  | x |
|  | Group 2 |  | x |  |  |  |  | x |
| **AARP** | Group 1 |  |  |  |  |  |  | x |
|  | Group 2 |  |  |  |  |  |  | x |
